# Navigating mental health in space: gut–brain axis and microbiome dynamics

**DOI:** 10.1038/s12276-025-01488-z

**Published:** 2025-06-30

**Authors:** Jiseon Yang, Hee-Dae Kim, Jennifer Barrila, Se-Hui Lee, Cheryl A. Nickerson, C. Mark Ott, Sharon A. Israel, Alexander Choukér, Jin-Young Yang

**Affiliations:** 1 Biodesign Center for Fundamental and Applied Microbiomics, Arizona State University, Tempe, AZ USA; 2 School of Life Sciences, Arizona State University, Tempe, AZ USA; 3 Department of Basic Medical Sciences, University of Arizona College of Medicine-Phoenix, Phoenix, AZ USA; 4 Department of Integrated Biological Sciences, Pusan National University, Busan, Republic of Korea; 5 Biomedical Research and Environmental Sciences Division, NASA Johnson Space Center, Houston, TX USA; 6 Independent Consultant, Industrial/Organizational Psychology, Miami, FL USA; 7 Laboratory of Translational Research Stress and Immunity, Department of Anesthesiology, LMU University Hospital, LMU Munich, Munich, Germany; 8 Institute for Future Earth, Pusan National University, Busan, Republic of Korea; 9 Department of Biological Sciences, Pusan National University, Busan, Republic of Korea

**Keywords:** Biological therapy, Mucosal immunology

## Abstract

Long-term space travel presents significant mental health challenges, with documented psychological and neurological impacts observed during spaceflight and in simulated space environments. Terrestrial studies have highlighted the connection between the human gut microbiome and neuropsychological health, known as the gut–brain axis. Recent research has demonstrated alterations in astronauts’ microbiomes during spaceflight. Here we explore interesting parallels between these microbiome changes in astronauts and those observed in terrestrial studies involving individuals experiencing psychological distress. Key areas of focus include how various space-related stressors impact the complex interplay between the gut microbiome, immune responses and mental health outcomes. This is illustrated by the effects of space radiation and circadian rhythm disruptions on the microbiome, as well as the roles of immune regulation and the integrity of gut and blood–brain barriers in shaping mental health outcomes through the gut–brain axis. Potential interventions and strategies for microbiome monitoring are suggested as critical to mitigating psychological risks during extended space missions. These findings underscore the need for further research to better understand how the microbiome responds to spaceflight environments and to develop targeted interventions that safeguard mental health during long-duration missions.

## Microbiome dynamics links space travel and psychological well-being

Space travel poses unique challenges, including increased risk of psychological conditions such as anxiety and depression. Researchers explore how spaceflight may impact mental health through changes in the gut microbiome, which is closely link to brain function. Data from astronauts aboard the International Space Station show microbiome shifts similar to those seen in individuals with mental health disorders on Earth, in human and animal studies. In this Review, the authors suggest space stressors—such as microgravity, radiation and circadian disruption—may alter gut microbial composition and host immune function, potentially affecting neuropsychological outcomes. The authors propose microbiome monitoring as a noninvasive method to detect early signs of mental health risks in space. Understanding microbiome dynamics and gut–brain interactions in spaceflight is crucial for safeguarding the mental health of astronauts during long-duration missions.

This summary was initially drafted using artificial intelligence, then revised and fact-checked by the author.

## Introduction

Astronauts undertaking space missions often face psychological challenges, such as anxiety, post-traumatic stress, sleep anomalies, acclimation difficulties and depression^1–6^. Many reports have described psychological difficulties experienced by crew members during space missions, and these challenges often become more pronounced during prolonged missions, indicated by reports from the 84-day Skylab 4 mission and the experiences of astronauts during extended stays on the International Space Station (ISS)^5^. Indeed, the early termination of the Soyuz T14 mission to the Salyut 7 Russian space station is believed to have been influenced by psychological difficulties, particularly depression^1^.

The National Aeronautics and Space Administration (NASA) has conducted the Lifetime Surveillance of Astronaut Health program and observed symptoms of anxiety and depression that meet diagnostic criteria indicated in the Diagnostic and Statistical Manual of Mental Disorders, during ISS spaceflights^6^. Using the Integrated Medical Model (IMM), 85.2% of female and 22.8% of male astronauts were estimated to meet the anxiety criteria, and 43.2% of female and 34.8% of male astronauts fulfill the depression criteria^6^. When considering generalizability, it should be noted that the IMM uses incidence rates based on terrestrial studies. Considering astronauts’ generally superior health compared with the public, these estimates highlight the significant, and cumulative, mental health impacts of space travel and the associated risks. Thus, it is vital to develop effective mitigation strategies when planning future long-term space missions. NASA has long recognized the stressors of space missions on the general health of astronauts, but understanding the impact of these stressors on psychological or neurological health has historically not received as much attention as other health categories. However, it has become a significant factor as NASA plans to transition from low Earth orbit to deep space exploration (for example, Moon and Mars), the latter of which is associated with extended durations in this stressful environment. Whereas the risks with psychological, cognitive or mental health are relatively lower in short-term space missions, the impact becomes greater during long-term missions. NASA provided the list of risks impacting human health and performance in space (https://humanresearchroadmap.nasa.gov/Risks/), which includes risk of adverse cognitive or behavioral conditions and psychiatric disorders; risk of performance and behavioral health decrements due to inadequate cooperation, coordination, communication and psychosocial adaptation within a team; risk of performance decrements and adverse health outcomes resulting from sleep loss, circadian desynchronization and work overload; and risk of spaceflight-associated neuro-ocular syndrome.

## Space stressors impacting physical and psychological health of astronauts

Astronauts are exposed to extreme environments in space such as space radiation and reduced gravity, along with disruptions to circadian cycles, all within an isolated and confined setting^6^. This harsh environment induces various physical and psychological hardships for astronauts that can further negatively impact neurological and psychological stability. Moreover, prolonged isolation far from Earth with limited resources (for example, lunar or Mars missions) can induce significant stress. The ISS orbits Earth 16 times a day, resulting in astronauts experiencing 16 sunrises and sunsets per 24-h period. This leads to an unnatural light–dark cycle that disrupts circadian rhythms, affecting hormonal regulation (for example, melatonin secretion, cortisol fluctuations and other stress hormones), metabolic processes (for example, glucose metabolism and insulin sensitivity), bone and muscle health, and cognitive function. Studies have demonstrated that astronauts often experience immune dysfunction, loss of muscle and bone density, and sleep deprivation, which can be neuropsychological stressors or can exacerbate psychological distress impacting mental stability and behavior^7–13^. Long-term spaceflight has been shown to result in severe molecular alterations in the expression of circadian clock genes, including *Per2*, *Cry2* and *Rorc*, particularly in skeletal muscle, indicating profound impact on physiological rhythms at the cellular level, beyond acute circadian misalignment^14^. Sleep deprivation and disruption of circadian rhythms are known to adversely influence hormone balance, which can compromise both physical health and mental resilience^15,16^. In addition, a lack of mechanical load due to reduced gravity can lead to a loss of muscle and bone density, resulting in critical impacts on physical activity, pain, calcium signaling, neurotransmitter levels and inflammation^17–19^. These factors act as significant stressors that contribute to neurological disturbances^17,18^. Moreover, these effects could be further exacerbated by exposure to radiation^20–22^, and there are numerous studies reporting adverse radiation effects on neurodegeneration, cognitive function and depressive symptoms^23–26^.

**Fig. 1:**
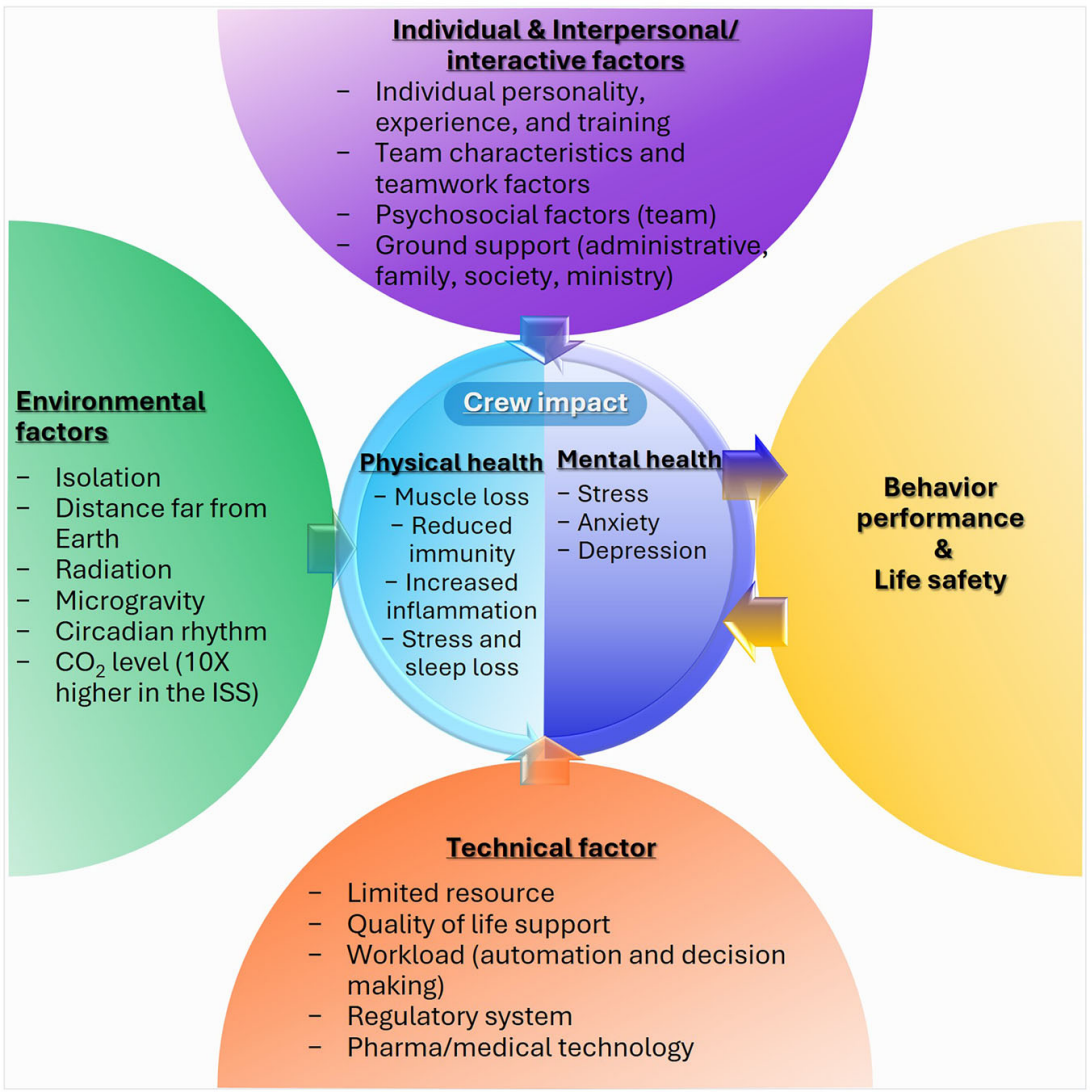
Schematic representation of risk factors affecting the physical and psychological health of astronauts during space travel. Examples of the multifaceted challenges of spaceflight and their impact on crew health and performance, which can shape overall crew health, behavior, performance and safety. Key factors include environmental stressors such as isolation and microgravity, interpersonal dynamics and technical limitations (for example, resource scarcity and life support systems). These interconnected risks collectively influence the physical and mental well-being of astronauts.

Neuropsychological challenges such as anxiety, depression, cognitive dysfunction, sensory and perceptual changes that astronauts experience during extended space missions are likely to be derived from a combination of environmental, individual, interpersonal/interactive and technical factors that significantly impact their physical and psychological well-being while in space (Fig. 1). Figure 1 highlights the interconnected risk factors affecting physical and psychological health of astronauts during space travel.

### Complex interplay of stressors in the space environment and the psychoneuroimmunological effects

**Fig. 2:**
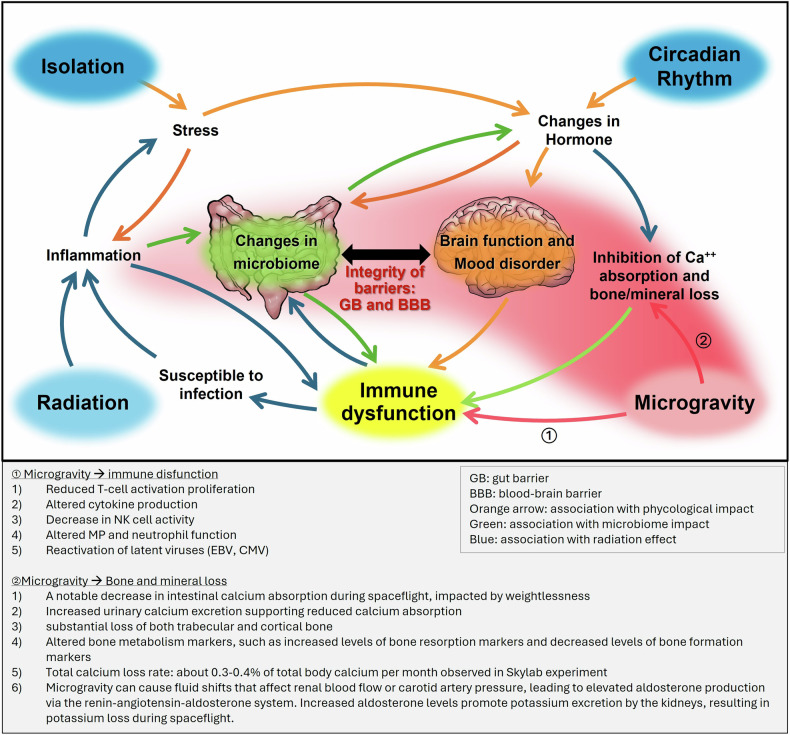
Schematic figures of the complex interplay of stressors in the space environment and the psychoneuroimmunological effects. The complex interplay between various spaceflight stressors and their effects on humans is shown. The arrows indicate the effects of these stressors and their influence on inflammation, hormone changes and barrier integrity (GBs and BBBs). Associations are color-coded: orange for psychological impact, green for microbiome impact and blue for radiation effects. The black arrow in the middle highlights the bidirectional relationship between changes in the microbiome and brain function.

Factors such as microgravity, radiation, nutrition, circadian rhythm disruption and psychological stress in space significantly affect immune function. These influences interact in complex ways, impacting neurological, psychological and immune systems in astronauts, which together shape their mental resilience and overall immune responses. This is known as psychoneuroimmunological effects, where interactions between the nervous, immune and psychological systems demonstrate how stress and emotions significantly affect physiological health. Figure 2 illustrates the complex interplay of stressors in the space environment and how they can impact crew mental health and how this, in turn, links to immunity. The diagram is a simplified representation of psychoneuroimmunological effects of spaceflight deliberatively designed to link the aspects of psychological stressors, neurological functions, immune responses and environmental factors in space. It illustrates how elements such as isolation, radiation, microgravity and circadian rhythm disruption collectively influence the microbiome, brain function and immune system, ultimately contributing to the mental resilience and overall health of astronauts (Fig. 2). The diagram emphasizes the link between the microbiome and brain function, highlighting the roles of gastrointestinal and blood–brain barrier (BBB) integrity. These psychoneuroimmunological alterations in space environments can further escalate the risks of infectious diseases and imbalances in the host–microbiome relationship, posing additional challenges to astronaut health.

During deep space missions, increased radiation exposure has the potential to impact astronaut physiology in ways that could alter psychological stress. Unlike low Earth orbit, deep space exploration is outside of Earth’s protective magnetic field and would result in astronaut exposure to higher levels and different types of radiation, including galactic cosmic radiation, consisting of high charge and energy (HZE) particles. Ionizing radiation is known to damage cells and tissues, as well as brain structure and the neurotransmission system^27^, posing a significant threat to the brain and cognitive abilities^28^. In terrestrial studies, ionizing radiation has been found to modify functions of various immune cells, including antigen-presenting cells, natural killer cells and T cells^29–32^, and can induce chronic neuroinflammation and other complications in the brain that negatively impact cognition^33^.

Recent studies reported that spaceflight affects human brain structure^34–44^, including changes in brain position, ventricular expansion, perivascular space, brain ventricular volume and white matter. These changes may align with shifts in functional behaviors, including posture control^40,45,46^, potentially leading to physical dysfunction, poor performance or adaptive plasticity associated with spaceflight^44,47^. The impact of microgravity on bone loss and musculoskeletal fitness is well known^48,49^. Reduced immunity and sleep loss in spaceflight are also well documented^9,50,51^ Collectively, these factors emphasize the need for comprehensive strategies to mitigate the neuropsychological impact of space travel on astronauts and the physiological burden, highlighting the need for further study and research in this area^52–56^. While space stressors such as microgravity, radiation and circadian rhythm disruptions have well-documented effects on astronaut health, their influence extends beyond direct physiological impacts. One emerging area of research highlights how these spaceflight conditions profoundly alter the human microbiome, which plays a crucial role in regulating immunity, metabolism and even neuropsychological function. The following section explores the significant shifts in microbial communities observed in astronauts during space missions.

## Human microbiome in space environments

Many studies have documented qualitative and/or quantitative alterations in the microbiome across various body sites such as gut, skin and saliva due to the effects of the space environment, highlighting the pronounce impact of spaceflight on microbial communities and host–microbe interactions^57–66^. For example, Lencner et al. investigated the intestinal microbiota of 24 cosmonauts, focusing on lactoflora dynamics before and after spaceflight, and found that prelaunch stress significantly disrupted *Lactobacillus* populations, with greater alterations after shorter missions, probably due to limited adaptation time and individual variability^62^. Similarly, another study of 12 cosmonauts identified consistent shifts in microbial composition, with significant reductions in *Bifidobacterium* and *Lactobacillus* during flight^64^.

In murine models, studies have shown significant impacts of spaceflight on the gut microbiome. A 37-day mission revealed increased microbial diversity and altered community structure, with reductions in *Bacteroidetes* and elevations in *Firmicutes*, resulting in a higher *Firmicutes*-to-*Bacteroidetes* ratio^63^. Specifically, *Tyzzerella* (a genus in the Lachnospiraceae family) and *Hydrogenoanaerobacterium* showed decreased abundance, whereas Ruminococcaceae *UCG-010* and other genera of the Lachnospiraceae family exhibited increased abundance in flight animals^63^. Liu et al. explored the gut microbiome of five astronauts across two spaceflight missions, lasting 15 and 35 days, respectively^61^. Fecal samples collected at multiple time points before and after flight revealed significant compositional and functional changes while retaining individual-specific microbial signatures^61^. Notably, an increase in the abundance of *Bacteroides* was observed, accompanied by a decrease in *Lactobacillus* and *Bifidobacterium* abundances during both missions^61^. Turroni et al. investigated the dynamics of the gut microbiota in a 520-day ground-based psychosocial isolation study intended to mimic a human mission to Mars (MARS500)^60^. They observed the resilience of individual microbiota profiles (individual specificity) within confined and controlled environments in a ground-based simulation. However, early increases in *Bacteroides* and reductions in *Lactobacillus* and *Bifidobacterium* suggested potential risks of inflammation and disruptions to host immune and metabolic homeostasis under prolonged stress and confinement^60^. Voorhies et al. examined the microbiome changes across various body sites of nine astronauts who spent 6–12 months in the ISS^59^. This study revealed that the crew microbiomes underwent significant changes in microbiome in the gastrointestinal tract, skin, nose and tongue, with a notable convergence in gut microbiota composition due to a decrease in certain bacterial taxa^59^. These alterations were linked to changes in cytokine profiles and correlated with increased skin sensitivity issues among crew members, highlighting the need for further research on the health implications of microbiome shifts during space travel^59^. In particular, the gastrointestinal microbiome displayed compositional changes with increases in proinflammatory taxa such as *Parasutterella* and decreases in health-promoting taxa, including *Akkermansia*, *Fusicatenibacter*, *Pseudobutyrivibrio*, *Ruminococcus* (Ruminococcaceae family), *Dorea* and members of the Lachnospiraceae family^59^. The reductions in these taxa were associated with decreased production of short-chain fatty acids and increases in proinflammatory cytokines, such as IL-8 and TNF-α, suggesting a potential link between microbiome alterations and immune dysregulation in space^59^. Further supporting this link, single-cell multi-omics profiling of the Inspiration4 crew provided additional evidence that spaceflight-induced microbiome shifts are associated with immune dysregulation^67^. The study observed an increase in *Bacteroides* and *Parabacteroides* after flight, coupled with suppressed MHC class I gene expression and elevated levels of proinflammatory cytokines^67^. These results suggest that microbiome changes may contribute to immune alterations during spaceflight, reinforcing previous studies on professional astronauts and murine models. Moreover, the study distinguished between transient microbiome changes that reversed after returning to Earth and more persistent alterations that lasted beyond the mission, indicating long-term implications of spaceflight exposure on astronaut health. For instance, alpha diversity increased in space but returned to preflight levels within 2 months, indicating a transient shift. However, certain bacterial taxa, such as *Akkermansia* and *Fusicatenibacter*, remained depleted after flight, suggesting potential long-term impact. Similarly, reductions in Proteobacteria (*Acinetobacter* and *Moraxella*) in the skin and nasal microbiome persisted beyond 60 days, classifying them as long-term alterations. Morrison et al.^58^ analyzed body swabs and saliva samples from four ISS astronauts using metagenomic sequencing and microarrays^58^. While overall microbial diversity remained stable, individual saliva analyses revealed significant changes, including increase in *Prevotella* and a decrease in *Neisseria*, *Rothia* and *Haemophilus*^58^. In addition, significant changes in antimicrobial resistance gene markers were observed in saliva samples^58^. Urbaniak et al.^57^ focused on the salivary microbiome of ten astronauts and found *Streptococcus* to be the most abundant genus in saliva, with its relative abundance decreasing during spaceflight^57^. At the phylum level, Proteobacteria and Fusobacteria show increased relative abundances during flight, while Actinobacteria demonstrates a significant decrease^57^. The increase in Fusobacteriaceae may correlate with shifts in immune responses or microbial interactions influenced by microgravity and confinement^57^. The NASA Twins Study revealed that, during spaceflight, the gut microbiome experienced taxonomic and functional shifts but largely returned to baseline after flight, while immune system changes showed inflammatory responses and stress responses but preserved core functionality^65^. Cognitive performance remained stable in-flight but declined after flight, with effects persisting for up to 6 months after return to Earth^65^. These findings highlight resilience in some systems and potential long-term risks in others during prolonged space missions. The first all-civilian, private crew mission to orbit demonstrated that spaceflight induces both transient and persistent microbiome changes, including the enrichment of gut-associated bacteria such as *Bacteroides* and *Parabacteroides* after flight, probably driven by dietary shifts and adaptations to space-specific stressors^66^. The summaries are presented in Table 1. While the exact causes of microbiome changes in space are not fully understood, a combination of factors such as microgravity, radiation, altered circadian rhythms and other altered individual physiological responses might have contributed to these changes in the space microbiome. Interestingly, many of these microbiome alterations observed in astronauts suggest a potential association with psychological stress and immune dysfunction on Earth, drawing attention to the potential role of the gut microbiome in mental health. This connection underscores the importance of examining terrestrial studies that have investigated the influence of microbiome on mental health, particularly its role in psychiatric conditions such as depression and anxiety.

**Table 1:** Notable spaceflight and spaceflight analog microbiome research.

Summary	Ref.
The gut microbiome of mice after a 37-day spaceflight, revealed increased microbial diversity and changes in microbial community structure associated with the space environment, suggesting significant impacts on energy metabolism and a potential link between host and microbial metabolism, highlighting the importance of the gut microbiome in mammalian health during spaceflight.	Jiang, P. et al.^63^
The gut microbiome of five astronauts across two spaceflight missions revealed significant changes in its composition and function, yet preserved individual-specific microbial signatures.	Liu, Z. et al.^61^
Prelaunch stress significantly impacted the lactoflora population of cosmonauts, with these effects more pronounced after shorter space missions owing to insufficient adaptation time and individual variations in response.	Lencner, A. A. et al.^62^
Research on the intestinal microflora of 12 cosmonauts before and after space missions revealed significant shifts in *Bifidobacterium* and lactobacilli during flight.	Liz’ko, N. N.^64^
The MARS500 project, representing the longest duration ground-based space habitat analog mission mimicking prolonged life sharing in a confined and controlled environment, revealed that the human gut microbiota remains dynamic and capable of adapting to long-term confinement in a controlled environment.	Turroni, S. et al.^60^
During long-term missions to the ISS, astronauts’ microbiomes indicated significant changes in the gastrointestinal tract, skin, nose and tongue, with a notable convergence in gut microbiota composition due to a decrease in certain bacterial taxa. These alterations were linked to changes in cytokine profiles and might have contributed to increased skin sensitivity issues among crew members.	Voorhies, A. A. et al.^59^
The study investigated the microbiome of four ISS astronauts, tracking microbial changes before, during and after their missions. While overall microbial diversity remained stable, individual saliva analyses revealed significant shifts, notably an increase in *Prevotella* and a decrease in *Neisseria*, *Rothia* and *Haemophilus*. Changes in antimicrobial resistance genes, particularly elfamycin resistance and a β-lactam marker, were observed.	Morrison, M. D. et al.^58^
An evaluation of ten astronauts indicated that, while *Streptococcus* was the most abundant bacterial species in saliva, its relative concentrations decreased during spaceflight. This finding and other shifts in microbial diversity indicated correlations between microbiome shifts and viral titers suggested interactions between oral microbiome and viral activity.	Urbaniak, C. et al.^57^
The NASA Twin Study included microbiome investigations analyzing changes in the gut microbiome of astronaut Scott Kelly during his year-long space mission, compared with his twin brother Mark Kelly on Earth, revealing shifts in microbial diversity and composition probably influenced by spaceflight conditions.	Garrett-Bakelman, F. E. et al.^65^
The first all-civilian, private orbital mission revealed spaceflight-induced microbiome changes, probably influenced by dietary shifts and space-specific stressors.	Tierney, B. T. et al.^66^

## Gut microbiome and mental health (terrestrial studies)

In recent years, a growing body of research has suggested a complex interplay between our gut microbiome and mental health^68–70^. Multiple studies have found associations between alterations in the gut microbiota and mental health disorders^71–76^. For example, Radjabzadeh et al. reported a significant association with depressive symptoms and gut microbiome through a large-scale research study involving 1054 participants from the Rotterdam Study cohort and an additional 1539 participants from the Amsterdam HELIUS cohort which comprised 6 different ethnic groups^75^. The authors found 13 microbial taxa significantly associated with depression, including the Ruminococcaceae (*Subdoligranulum*, Ruminococcaceae UCG-002, UCG-003 and UCG-005, *Ruminococcus gauvreauii* group and Ruminococcaceae), Lachnospiraceae (*Coprococcus, Sellimonas, Lachnoclostridium* and Lachnospiraceae UCG-001) *Eggerthella*, *Hungatella* and *Eubacterium ventriosum*^75^. The most important bacterial genus in predicting depressive symptoms was identified to be Ruminococcaceae UCG-005, and other significant predictors of depressive symptoms included Christensenellaceae, *Lachnoclostridium*, *Eggerthella*, *Sellimonas*, *Hungatella*, *Roseburia*, *Streptococcus*, *Bacteroides*, *Anaerotruncus*, *Dorea*, *Blautia*, *Veillonella*, *Desulfovibrio*, *Anaerostipes* and *Bifidobacterium*^75^. Interestingly, many of these bacteria (including *Coprococcus*, *Lachnoclostridium*, Ruminococcaceae, Lachnospiraceae*, Roseburia* and *Eubacterium*) are known to be involved in the production of butyrate, a short-chain fatty acid associated with a number of health benefits including improved gut health, reduced neuroinflammation, BBB integrity and protection against depression^77–82^. Another study examined 76 fecal samples from 46 patients with major depressive disorder and 30 healthy control participants^69^. The authors found a significant difference in microbiome composition between the patients with major depressive disorder and the controls at the family level including Lachnospiraceae, Prevotellaceae, Ruminococcaceae, Veillonellaceae, Acidaminococcaceae, Fusobacteriaceae and Porphyromonadaceae^83^. Those families also included the bacteria that are known to produce butyrate or gamma-aminobutyric acid (GABA). A separate study investigating gut microbiome of patients with bipolar disorder showed significant differences in *Faecalibacterium*, Actinobacteria, Coriobacteria and Ruminococcaceae^74^.

Rodent studies have provided supportive evidence suggesting that alterations in the gut microbiota can impact mouse behavior associated with depression, by affecting neurotransmitter levels, immune system function and other factors that influence mood^84^. *Coprococcus*, *Pseudobutyrivibrio* and *Dorea* were separately reported to be associated with stress-induced changes^85^. There are also several reports demonstrating that fecal microbiota transplantation from either patients with depression or mice with behavioral disorders into healthy animals caused depressed behaviors^72,86,87^. These studies identified a significant difference in the relative proportions of *Prevotella* (Prevotellaceae family), *Dialister* (Veillonellaceae family), *Eggerthella*, *Holdemania*, *Gelria*, *Turicibacter*, *Paraprevotella* (Prevotellaceae family) and/or *Anaerofilum* between the patients (or animals) with depression and healthy controls, indicating the link between gut microbiome and neurologic dysfunction. Also, another study examined an impact of traumatic brain injury on the gut microbiota in mice^88^. The study reported a decrease in butyrate-producing bacteria, particularly *Eubacterium ventriosum*, along with reductions in *Lactobacillus gasseri* and *Ruminococcus flavefaciens* after traumatic brain injury^88^, suggesting the potential association between the decrease in these organisms and the post-injury depression and anxiety-related responses. Overall, whether studied in humans or animals, many bacteria associated with mental health disorders and brain functions are known to be involved in the synthesis of key neurotransmitters such as glutamate, serotonin, GABA, short-chain fatty acids or hormones that can influence brain function and behavior^77–82,89–92^.

Although specific changes in the gut microbiome associated with mental health vary across studies, the intricate connection between the gut microbiome and mental health underscores its influence on systemic physiological processes, including mood regulation, immune responses and brain function^69,70,93–95^. This interplay, mediated through the gut–brain axis, is critical for neurotransmitter production (serotonin and GABA), immune modulation and metabolic pathways, including the synthesis of short-chain fatty acids and hormones, all of which are highly susceptible to environmental stressors^69,70,93–95^. Environmental stressors such as radiation exposure and disruptions of circadian rhythms are particularly significant in space, where they have been shown to substantially influence immune function, hormonal balance and microbial community composition^96–98^. These challenges can further complicate metabolic regulation and mental health^69,70,93–95^.

Terrestrial studies showed that ionizing radiation from medical treatments, such as cancer radiotherapy, can alter microbial diversity and abundance^99,100^. Radiation-induced damage to the intestinal lining shifts gut microbial composition, disrupting immune functions such as cytokine production, inflammation regulation, wound healing and susceptibility to infections^99,100^. Similarly, space radiation, characterized by its intensity and complexity from high-energy cosmic rays and solar particles, presents unique and intensified challenges for astronauts. Studies of spaceflight-associated alterations in microbiome are described in the next section, focusing on pronounced changes in microbial diversity and their association with potential psychological health risks during extended space missions.

In space, the absence of a conventional day–night cycle disrupts circadian rhythms, which are vital for regulating hormonal balance and immune system function^96,97,101–103^. This disruption can amplify stress responses, alter hormone levels such as cortisol and melatonin, and negatively impact the gut microbiome. These changes are believed to influence metabolism and immune responses as well as body responses to stresses such as radiation^96,97,101–103^. For instance, studies in mice subjected to abnormal light cycles revealed alterations in gut microbial composition and increased susceptibility to radiation, underscoring the critical interplay between circadian rhythms and microbial populations in maintaining health^103^.

Both radiation exposure and circadian rhythms disruptions contribute to cascading effects on stress, mood and overall health. Immune dysfunction is a well-documented consequence of spaceflight across both short- and long-duration missions^7–12^. Stress hormones, such as cortisol, regulated by circadian rhythms, can directly influence gut microbial composition, potentially exacerbating immune dysregulation and mood disturbances. The interconnected relationship between microbiome health, immune function and psychological well-being creates a feedback loop, where microbial imbalances may amplify stress and mood disorders, further compromising astronaut health. Therefore, strategies to mitigate the adverse effects of space radiation and circadian rhythm disruptions on the microbiome and maintaining microbiome health in space is critical. The relationship of the microbiome with immune responses is discussed further below. The growing body of terrestrial research suggests that gut microbial composition is intricately linked to neurochemical balance, inflammation and overall mental health. These findings raise an important question: what mechanisms mediate this gut–brain connection? The next section delves into the gut–brain axis and immune interactions, which serve as key regulators of mental well-being and may offer critical insights into astronaut health.

## Potential gatekeepers of mental health: gut–brain axis and immune interactions

### Interactions in the gut–brain axis and immune function

The gut microbiome plays a significant role in shaping human neurophysiological processes, under both normal and disease conditions^104^. The intricate relationship between the gut microbiome and mental health in brain function is often described as the gut–brain axis, a complex and dynamic bidirectional communication network that links the central nervous system with the gastrointestinal tract^68–70,93–95,104^.

Various mechanisms are believed to operate within gut–brain axis communication, each contributing to the intricate interaction between the gut and the brain. This axis involves direct neural pathways, such as the vagus nerve, which serves as a crucial conduit for transmitting signals from the gut to the brain and vice versa^105^. In addition, the gut–brain axis is mediated by biochemical signaling, where gut-derived metabolites, such as neurotransmitters and short-chain fatty acids, along with immune system interactions, play a pivotal role in influencing brain function and behavior^106–109^. These interactions can affect various aspects of mental health, including mood, stress response and cognitive functions, highlighting the potential for therapeutic interventions targeting the gut–brain axis to treat or manage neurological and psychiatric disorders.

Short-chain fatty acids produced by gut microbiota have been shown to enhance mental health and cognitive function, with studies indicating reductions in anxiety and depressive behaviors^106^ as well as improvements in learning and memory tasks^110–112^. Certain gut bacteria metabolize amino acids to produce neurotransmitters such as GABA, serotonin and dopamine, which are essential not only for gut function but also for regulating mood and behavior through their effects on the brain^113,114^. Moreover, the gut microbiome can influence neurotransmission by altering the expression of neurotransmitter receptors^115^. Beyond these effects, numerous studies have shown that gut microbiota impact various neurological processes, including neurogenesis, microglial activation and synaptic plasticity, under both normal and stressful conditions^104,116,117^.

While the influence of gut microbiota on neuronal functions is well established, the gut–brain axis is equally crucial in regulating the immune system. The interaction between gut microbes and the immune system plays a vital role in shaping normal brain functions and mental health, further intertwining gastrointestinal and central nervous system functions in a complex, interdependent relationship^118^. The gut microbiome modulates immune activity, and therefore, disruptions in this immune regulation can impact brain function, potentially contributing to mood disorders such as depression^68,69,94^. In a model of chronic gastrointestinal inflammation, modulation of gut microbiota successfully normalized anxiety-like behavior associated with the inflammation^119^. The gut microbiome can also directly affect the operation of immune cells^89^, such as T cells^120^ and B cells^121^. In the brain, gut microbiota can modulate the activity of microglia, the primary immune cells of the central nervous system, influencing the stress response^122^.

A growing body of evidence supports that chronic stress can lead to inflammation and immune dysfunction, which are significant contributors to mood disorders^123–125^. Numerous studies have reported elevated levels of circulating proinflammatory markers such as circulating C-reactive protein, interleukin (IL)-6, IL-1 and tumor necrosis factor alpha (TNF-α) in patients with depression^124,126^. Moreover, recent research has illustrated how body-to-brain inflammatory spread, due to changes in brain inflammatory signaling, can cause depressive behavior^127^. This process involves inflammatory cytokines activating the NF-kB signaling pathways in the brain, leading to depressive-like behavior, and the complex interplay between glucocorticoid receptors and NF-kB signaling further modulating these effects. Emerging studies have identified specific immune markers, such as MMP8, derived from circulating monocytes, as reliable indicators of depression, supported by both preclinical and clinical data^128^. The study identified elevated MMP8 as a key factor in depression and stress-related behavior changes, with its depletion reducing these effects, suggesting MMP8 as a potential therapeutic target.

### Gatekeeping of the gut–brain communications: GB and BBB

With the understanding that circulating metabolites and immune factors play a significant role in the gut–brain axis and its influence on mood disorders, it becomes critical to explore how these elements can be modulated. The barrier systems, specifically the gut barrier (GB) and the BBB, are central to this modulation^129^. These barriers regulate the passage of metabolites and immune factors between the gut and the brain, maintaining the integrity of the gut–brain axis. Disruptions in these barriers can increase permeability, allowing inflammatory mediators and pathogens to cross into the brain, exacerbating neurological and psychiatric conditions. Therefore, targeting the integrity and function of these barrier systems offers a promising avenue for therapeutic interventions aimed at modulating the gut–brain axis and mitigating mood disorders.

Two of these barriers, GB and BBB, have recently attracted significant attention from researchers. This focus originates from the potential link between these barriers and the pathophysiological mechanisms underlying psychiatric disorders^107,130–132^. The GB and BBB share similarities in their composition, particularly in the presence of tight junction proteins such as occludins and claudins^107^. These barriers play a crucial role in influencing brain functions, often in conjunction with immune and cardiovascular systems. As such, any alterations in these barriers alongside circulating inflammatory factors, especially in unique environments like space, could potentially impact brain function and manifest as psychiatric symptoms.

The GB serves as a primary line of defense and has a strong association with gut microbiomes. A malfunctioning GB can lead to adverse inflammatory effects on the body, including the brain, which are often attributed to disturbances in the gut microbiome^131^. Indeed, stress-induced activation of the hypothalamic–pituitary–adrenal (HPA) axis and subsequent secretion of glucocorticoids change the permeability of the GB. In chronically stressed female mice, stress disrupts GB integrity, leading to increased levels of circulating lipopolysaccharide-binding protein and subsequently increasing anxiety- and depression-like behaviors^129^. Chronic stress also reduces claudin-1, an intestinal epithelial tight junction protein, via glucocorticoid receptor-mediated transcriptional repression^133^. Conversely, butyrate appears to enhance intestinal barrier functions^134^.

Meanwhile, the BBB acts as a highly selective filter in the brain, allowing only specific nutrients and chemicals to pass from the circulatory system into the brain. Recent studies demonstrated that a compromised BBB, often referred to as a leaky BBB, can induce depression-like behaviors in mouse models subjected to chronic stress^135–137^. This leakiness was attributed to reduced expression of claudin-5, a component of tight junction protein complexes, and was also observed in human patients with major depression^137,138^. Notably, knockdown of claudin-5 alone is sufficient to induce depression-like behaviors, potentially allowing the passage of inflammatory cytokines (for example, IL-6) and infiltration of immune cells (for example, monocytes and activated T cells), which may induce behavioral deficits^107,137,138^.

Overall, maintaining the integrity of the GB and BBB is crucial for preventing stress-related inflammatory and mood disorders. Given these findings, it is reasonable to hypothesize that any changes in the functionality of these biological barriers or related vascular system, especially in the aforementioned challenging environment of space such as radiation and microgravity, could have significant implications for brain function and psychiatric health. While the precise mechanisms of environmental effects in space remain elusive, physical exercise stands out as a promising modifiable factor that can positively impact these barriers^107^. Exercise enhances neuronal activity in reward-processing brain areas^139^ and promotes an anti-inflammatory cytokine profile^140^. It strengthens the BBB by increasing astrocyte density^141^ and aquaporin-4 levels^142^, protecting barrier integrity. Exercise also benefits the GB, reducing inflammatory gene expression and boosting anti-inflammatory factors and antioxidants in intestinal immune cells^140^. These changes suggest that exercise-induced microbiome and systemic health improvements may offer therapeutic potential for mental health and well-being in the stressful space environment as well. Given the established links between the gut microbiome, immune function and mental health, alterations in the microbiome during spaceflight may have significant implications for astronaut psychological resilience. The following section explores these potential microbiome-mediated risks, drawing parallels between microbiome changes observed in astronauts and those linked to mental health conditions in terrestrial studies.

## Gut microbiome in space and mental health considerations

**Fig. 3:**
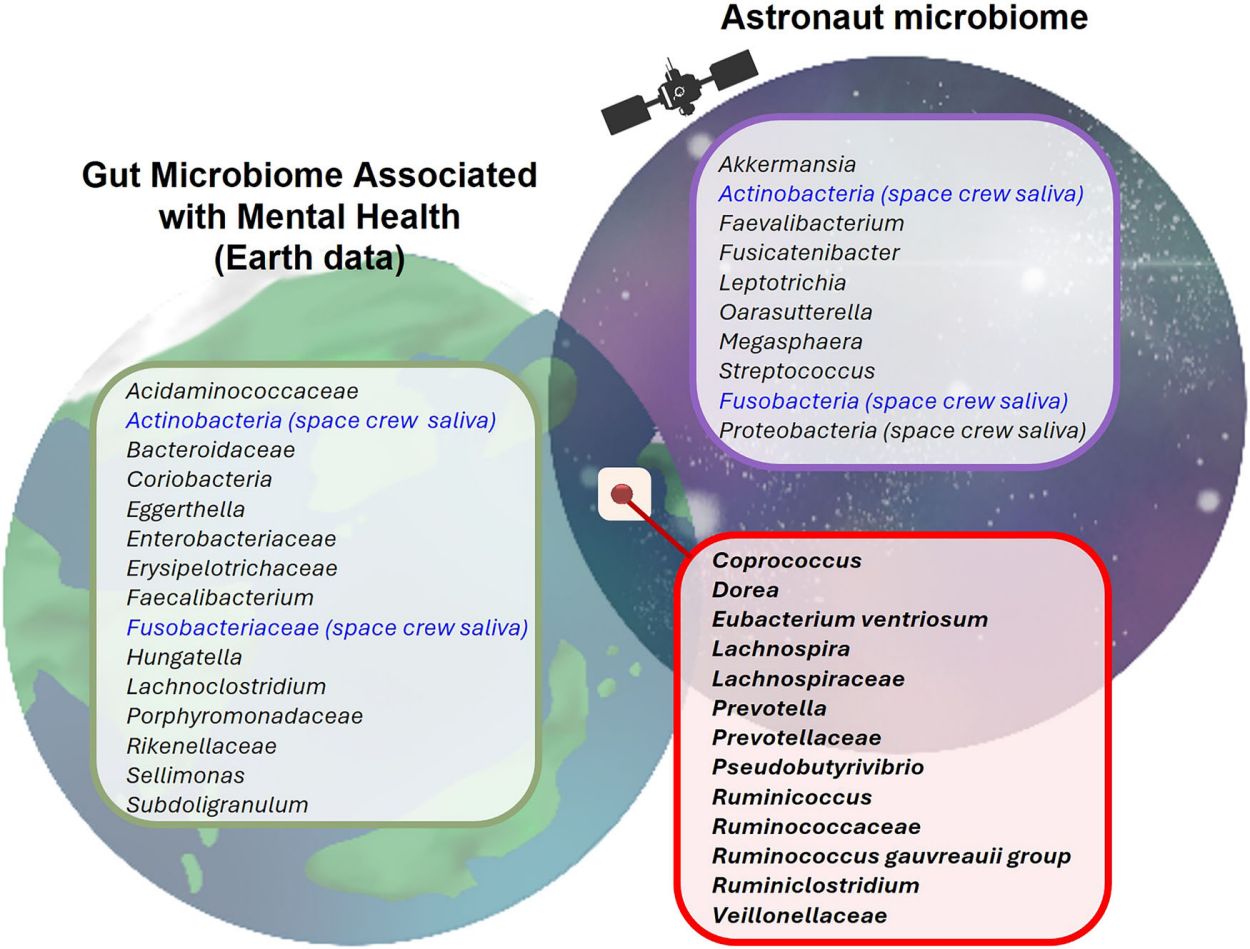
Comparison of astronaut microbiome and terrestrial microbiome influencing neuropsychological health. Bacterial taxa in blue represent those found specifically in space saliva samples, while other colors indicate shared or distinct microbial groups between Earth and space environments. The red box highlights bacterial groups that are specifically enriched or uniquely present in space, emphasizing shifts in microbial populations under spaceflight conditions.

During our literature searches, we observed some interesting parallels in the microbiomes reported for patients with mental health disorders and changes occurring in the gut microbiomes of nine astronauts who spent 6–12 months aboard the ISS^59^. In this study, the microbial composition of fecal samples collected at ten different time points before, during and after the mission (starting 240 days before launch and up to 60 days after landing). The study identified 17 gastrointestinal genera whose abundance significantly changed in space, including *Megasphaera*, *Ruminococcus*, *Leptotrichia*, *Veillonella*, *Prevotella*, *Akkermansia*, *Coprococcus*, *Eubacterium ventriosum* group, *Pseudobutyrivibrio*, *Fusicatenibacter*, *Streptococcus*, *Dorea*, *Ruminiclostridium*, *Faecalibacterium*, *Parasutterella*, *Lachnospira*, *Lachnospiraceae* ND3007 group and unclassified species^59^. Interestingly, about 11 genera among the 17 were previously reported to be associated with human neuropsychological health in terrestrial-based studies^72,75,83–88^ Moreover, *Ruminococcus* and Lachnospiraceae, which showed significant alterations during spaceflight, have also been identified in multiple terrestrial studies as being significantly associated with depression^59,60,75,83^. Specifically, *Ruminococcus*, which was reduced during spaceflight in the astronaut, has been link to decreased levels in patients with depression^71,75,83^. Similarly, an increase in Lachnospiraceae, which was significantly more abundant during spaceflight compared with preflight levels, has been linked to elevated depression levels in terrestrial studies^71,75^. However, some studies have reported conflicting results, linking reduced Lachnospiraceae levels to depression^83^. *Eubacterium ventriosum* group, *Ruminococcus* (Ruminococcaceae family), Lachnospiraceae, *Coprococcus*, *Ruminiclostridium* and Prevotellaceae were found to be significantly altered in patients with depression (Rotterdam study, *n* = 1054; HELIUS study, *n* = 1539), and random forest analysis with these large-scale human data reported that *Ruminococcus*, *Coprococcus*, Lachnospiraceae (which includes *Lachnospira*), *Veillonella*, *Streptococcus* and *Dorea* can be significant predictors of depressive symptoms^75^. Veillonellaceae, Lachnospiraceae, *Faecalibacterium* and *Prevotella*, along with *Ruminococcus*, were also reported to be negatively correlated with depressive symptoms^83^. Using animal models, *Coprococcus*, *Dorea* and *Pseudobutyrivibrio* were reported to be associated with those diagnoses, and *Eubacterium ventriosum* was suggested to be an important contributor to developing depression in a mouse study^88^. Changes in genera such as *Prevotella*, *Veillonella*, *Actinobacteria* and *Fusobacterium* previously linked to mental health in Earth-based mental health microbiome research were also observed in saliva of astronauts^57,58^. Overall, *Eubacterium ventriosum*, *Ruminococcus* (Ruminococcaceae family), Lachnospiraceae (family of *Lachnospira*), *Coprococcus*, *Ruminiclostridium*, Prevotellaceae, Veillonellaceae, *Faecalibacterium*, *Dorea* and *Pseudobutyrivibrio* exhibited changes during spaceflight and also in patients or animal models with psychological or behavioral disorders (summarized in Fig. 3). Many of these bacteria are known to be butyrate or GABA producers^83^. Microorganisms in the gut can produce and respond to the same neurochemicals that the brain uses to regulate mood and cognitive function, such as serotonin, dopamine and GABA^95,143^. In particular, it has been reported that *Ruminococcus gnavus* mediates the catabolism of dietary phenylalanine and tryptophan to generate phenethylamine and tryptamine, which directly stimulate serotonin biosynthesis in intestinal enterochromaffin cells via a mechanism involving activation of trace amine-associated receptor 1 (TAAR1) (serotonin metabolism)^143^. Studies showed that changes in the composition of the gut microbiota have been associated with various mental health disorders, including anxiety, depression, autism spectrum disorder and schizophrenia. However, while these associations have been found, the causality and mechanisms underlying these links are not fully understood.

### Potential use of probiotics as an intervention for mental health

Recent studies suggest that probiotics may have a positive impact on mental health. Certain probiotics, known as psychobiotics when used for mental health benefits, have been found to reduce symptoms of depression and anxiety in both humans and animals^70,144^. In animal studies, probiotic treatment was found to decrease anxiety and depression-like symptoms^145–149^, and it has been shown that some strains of *Lactobacillus* and *Bifidobacterium* have the ability to produce GABA^150^. Oral supplements with *Bifidobacterium longum* were found to normalize mouse anxiety-like behavior induced by gut chronic inflammation^148^, and ingestion of *Lactobacillus* strain regulated emotional behavior and central GABA receptor expression in mice^145^. *Bifidobacterium infantis* was found to regulate the exaggerated stress response in germfree mice, specifically by reversing the elevated levels of certain stress hormones (such as corticosterone) observed in response to physical restraint^151^. In addition, oral administration of *Lactobacillus helveticus* and *Bifidobacterium longum* in rats reversed behavioral effects of myocardial infarction, reducing depression-like symptoms and improving social interaction^152^. The intricate relationship between the gut microbiome and mental health, as uncovered by a plethora of studies, underscores the multifaceted role of the microbiome in human health. As we expand our exploration beyond Earth, understanding the behavior and function of the microbiome in space environments could become a new frontier, opening doors to challenges and opportunities for human adaptation and well-being in extraterrestrial habitats.

## Conclusion and perspectives

Space travel presents unique psychological and physiological challenges, emphasizing the critical need for comprehensive health monitoring. This Review highlights intriguing similarities between the gut microbiomes of astronauts who spent 6–12 months on the ISS and those of individuals with mental health diagnoses on Earth. The gut microbiome influences mental health by interacting with neurochemicals that regulate mood and cognitive function, and shifts in microbiota composition have been linked to disorders such as anxiety and depression. However, the underlying mechanisms remain unclear. Understanding these dynamics in space environments presents new challenges and opportunities for supporting human health in extraterrestrial habitats.

Given the potential for microbiome shifts in space to affect mental health, further investigation into its role in space, particularly its relationship with immune function and mental health, is essential. The interplay between the gut microbiome, the brain and the immune system in space-specific conditions—such as microgravity, radiation and other stressors—remains largely unexplored. Advancing our understanding of how these systems interact in spaceflight is crucial. This knowledge will not only deepen our understanding of the role of microbiome in neuropsychological function but also help to develop effective risk mitigation strategies, ultimately supporting long-term astronaut health and human adaptation to space.

**Fig. 4:**
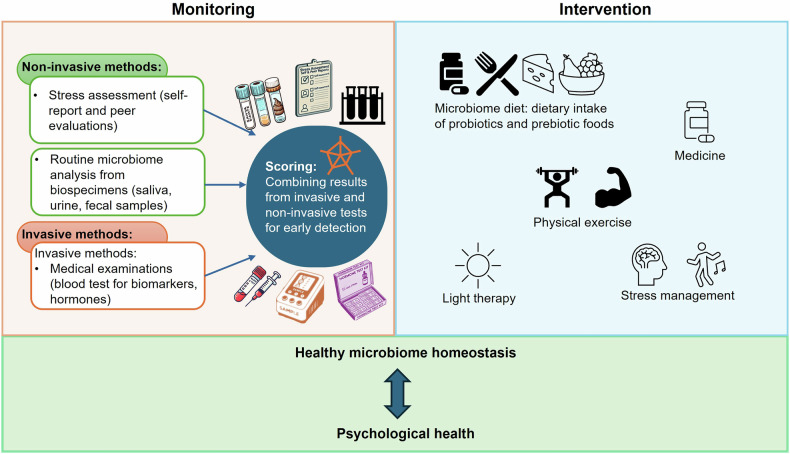
Monitoring and intervention strategies for maintaining microbiome homeostasis and psychological health. Monitoring involves noninvasive methods such as stress assessments and microbiome analysis from biospecimens, and invasive methods such as blood tests. Intervention strategies include dietary changes, medicine, exercise, light therapy and stress management, aiming to support healthy microbiome homeostasis and psychological well-being.

To address these challenges, we propose incorporating routine microbiome testing as a noninvasive tool for early detection of neuropsychological risks in astronauts. By combining microbiome analysis with psychological assessments (self-report or peer) and blood tests, early indicators of physiological or cognitive imbalance may be identified (Fig. 4). Invasive methods, such as blood analyses for biomarkers and hormones, can further enhance these insights, providing a more comprehensive understanding of both physical and psychological health. Integrating noninvasive and invasive methods may facilitate earlier detection and management of health conditions that are otherwise difficult to diagnose in the space environment.
